# Relationships Between Job Stress, Psychological Adaptation and Internet Gaming Disorder Among Migrant Factory Workers in China: The Mediation Role of Negative Affective States

**DOI:** 10.3389/fpsyg.2022.837996

**Published:** 2022-03-22

**Authors:** He Cao, Kechun Zhang, Danhua Ye, Yong Cai, Bolin Cao, Yaqi Chen, Tian Hu, Dahui Chen, Linghua Li, Shaomin Wu, Huachun Zou, Zixin Wang, Xue Yang

**Affiliations:** ^1^Longhua District Center for Disease Control and Prevention, Shenzhen, China; ^2^Faculty of Medicine, JC School of Public Health and Primary Care, The Chinese University of Hong Kong, Hong Kong, Hong Kong SAR, China; ^3^School of Public Health, Shanghai Jiao Tong University School of Medicine, Shanghai, China; ^4^School of Media and Communication, Shenzhen University, Shenzhen, China; ^5^Shenzhen Health Development Research and Date Management Center, Shenzhen, China; ^6^Guangzhou Eighth People’s Hospital, Guangzhou Medical University, Guangzhou, China; ^7^School of Public Health (Shenzhen), Sun Yat-sen University, Shenzhen, China; ^8^Kirby Institute, University of New South Wales, Sydney, NSW, Australia

**Keywords:** Internet gaming disorder, job stress, psychological adaptation, negative affective states, factory workers, China

## Abstract

Factory workers make up a large proportion of China’s internal migrants and may be highly susceptible to job and adaptation stress, negative affective states (e.g., depression and anxiety), and Internet gaming disorder (IGD). This cross-sectional study investigated the relationships between job stress, psychological adaptation, negative affective states and IGD among 1,805 factory workers recruited by stratified multi-stage sampling between October and December 2019. Structural equation modeling (SEM) was conducted to test the proposed mediation model. Among the participants, 67.3% were male and 71.7% were aged 35 years old or below. The prevalence of probable depression, probable anxiety, and IGD was 39.3, 28.7, and 7.5%. Being male, younger age, and shorter duration of living in Shenzhen were associated with higher IGD scores. Job stress was significantly associated with IGD (β = 0.11, *p* = 0.01) but not with negative affective states (β = 0.01, *p* = 0.77). Psychological adaptation was significantly associated with negative affective states (β = −0.37, *p* < 0.001) but not with IGD (β = 0.09, *p* > 0.05). Negative affective states were positively associated with IGD (β = 0.27, *p* < 0.001). The indirect effect of psychological adaptation (β = −0.10, *p* = 0.004) but not job stress (β = 0.003, *p* = 0.77) on IGD through negative affective states was statistically significant. The observed psychological correlates and mechanisms are modifiable, and can inform the design of evidence-based prevention programs for depression, anxiety, and IGD in this population.

## Introduction

As Internet game gains growing popularity worldwide, it poses an increasing threat to population mental and behavioral health (e.g., suicidal ideation, insomnia, cognitive impairment, sedentary lifestyle, violence). Internet gaming disorder (IGD) was defined as “persistent and recurrent use of the Internet to engage in games, often with other players, leading to clinically significant impairment or distress” by the fifth edition of the Diagnostic and Statistical Manual of Mental Disorders (DSM-5; p. 795) American Psychiatric Association [APA] (2013). It was defined as a mental disorder in the 11th Revision of the International Classification of Diseases (ICD-11).

Internet game is very popular among young people in China. Since 2015, China was the greatest game market in the world. As of 2019, it generated a total of 36.5 billion revenue which constituted 24.5% of global game revenue (Newzoo, 2019). Within 10 years, the total number of video game users in China grew ninefold, reaching 640 million in 2019 (Game Committee of the Publishers Association of China [GCPAC], 2019), accounting for 25.6% of global gamers (Newzoo, 2019). Along with the popularity may come greater susceptibility to addiction. Young people especially in Eastern Asia have been reported to be at great risk of having IGD (Paulus et al., 2018).

Migrant factory workers in China may be highly susceptible to IGD as this population tends to be relatively young, come from rural areas, have low education levels and health literacy, live with a low income, suffer from great stress, and have few coping resources (Solinger, 1999; Gu et al., 2006; Li et al., 2006; Koo, 2012). Many factories in China may not provide a supportive setting for healthy lifestyle and mental health, as the majority of their workers spend long hours working in a confined environment and most of them live in the factories’ dormitories. High prevalence of sedentary lifestyle and smoking has been evident in this population (e.g., Liu et al., 2015, 2016). Although Internet gaming is typically less harmful than many other behaviors, such as tobacco, alcohol and drug use, which are often used to cope with stress and regulate negative emotions (Corbin et al., 2013), excessive Internet gaming is harmful and may pose risks for vulnerable individuals and lead to IGD and other severe mental health problems (King et al., 2019). No study has investigated prevalence or factors of IGD in this population.

Factory workers make up a large proportion of China’s internal migrants. It is estimated that there is a total of more than 80 million such workers (National Bureau of Statistics of China [NBSC], 2019) and most of them are living and working in large coastal Chinese cities, such as Shenzhen, Guangdong Province (National Bureau of Statistics of China [NBSC], 2012, 2019). About 22.22% of the population in Guangdong province was made up of internal migrant workers (Bureau of Statistics of Guangdong Province [BSGP], 2019). With the large population size, their health-risk behaviors and mental disorders may contribute substantially to the burden of public health care system. Health professionals thus need to pay particular attention to IGD in this population (Biao, 2004).

Job stress is prevalent and may be a significant risk factor of IGD among migrant factory workers. Adopting the person-environment fit theory, common sources of job stress include workload (i.e., work-over-load resulting from time pressure), role conflict (i.e., having logically incompatible demands made upon the individual by two or more persons whose jobs are functionally interdependent with the individuals’ job), role ambiguity (i.e., a state in which a person has inadequate information to perform their role in an organization), and under-utilization of skills (i.e., under-utilization of previously acquired skills in carrying out tasks required on the job) (Harris et al., 1999). Such stress has been implicated in the etiology of poor mental health and psychosomatic disease among factory workers (e.g., House et al., 1979; Kawakami et al., 1992). Internet gaming may be handy and inexpensive for factory workers to cope with their job stress. As Griffiths (2002) suggested, convenience and affordability may be important factors of using online applications. Moreover, lack of alternative coping resources in addition to online applications may lead to excessive use or over-reliance on these online applications as a stress coping means, which will increase the risk of IGD (King et al., 2020). Thus, IGD can be seen as an adverse health and behavioral consequence of job stress coping. We did not locate any studies testing the association between job stress and IGD, although we found two studies reporting the relationships between general stress and IGD and between job stress and Internet addiction (Chen et al., 2014; Yu et al., 2018). It is warranted to investigate their levels and relationship among migrant factory workers, as such information facilitates planning programs for stress reduction and IGD prevention.

Psychological adaptation may be a protective factor of IGD among migrant factory workers. Based on the model of Searle and Ward (1990), psychological adaptation refers to how comfortable and happy a person feels with respect to being in the new culture, or anxious and out of place. It is a well-documented protective factor of maladaptive emotions and mental/behavioral problems, including depression, anxiety, smoking, drinking, and substance use, among migrants (Gonidakis et al., 2011; Curtis et al., 2018; Shi et al., 2019) and Chinese migrant workers (Qiu et al., 2011). Migrant workers may lead a stressful life as they are away from home, need to adapt to their new urban lifestyle (Berry, 1997; Ward et al., 2001), and be lack of social support and stress-coping resources (Liu et al., 2015). Maladaptation inevitably leads to psychological, behavioral, and social problems, while positive adaptation may imply great social support and positive coping responses to a new culture (Berry et al., 1994). There is, however, no study looking at association between psychological adaptation and IGD.

Negative affective states, such as depression and anxiety, are prevalent among migrant factory workers (Zhong et al., 2018), and are well-documented risk factors of IGD in young populations (e.g., Paulus et al., 2018; Wu et al., 2018; Bonnaire and Baptista, 2019). They may play as a mediator in the relationships between job stress/psychological adaptation and IGD. The general strain theory (Agnew, 1992) may support this mediation hypothesis. Agnew’s focus on negative affective states offers a general explanation of maladaptive behaviors; that is, strain related to external environment leads to negative affective states, and maladaptive behaviors may serve as a means of relieving such states (Agnew, 1992). This theory is one of strain theories and particularly focuses on how stressful environment or events influence behavior. This theory has been applied to explain how individuals who perceive external stress (e.g., perceived gender role stress, perceived academic stress) would develop addictive use of social networking sites and Internet through negative affective states, such as stressful feeling, depression, and anxiety (Yang et al., 2018). No study has applied this model to explain IGD among migrant factory workers.

The present study aims to test prevalence and potential factors of IGD among migrant factory workers. Furthermore, this study aims to examine a mediation model based on the framework of general strain model among migrant factory workers. It is hypothesized that job stress would be positively associated with (1a) negative affective states and (1b) IGD; psychological adaptation would be negatively associated with (2a) negative affective states and (2b) IGD; (3a) job stress and (3b) psychological adaptation would be indirectly associated with IGD through negative affective states.

## Materials and Methods

### Study Design

We conducted a cross-sectional survey in Longhua District of Shenzhen, China from October to December 2019.

### Participants and Data Collection

As high as 34.3% of the residents of Shenzhen are factory workers (Jun and Choi, 2015; Statistics Bureau of Shenzhen Municipality [SBSM], 2019). There are 1,805 factories and 513,215 factory workers in Longhua District. A stratified multi-stage sampling approach was used for recruitment: 16 factories were randomly selected by the research team, including four mechanical processing plants, three electronic devices manufacturers, three printing and dyeing factories, two chemical raw materials plants, one smelter, one garment factory, one food and beverage manufacturer, and one other factory; three to four workshops were then randomly selected from each factory. All full-time employees aged ≥18 years in the selected workshops were invited to participate in the study. Interested participants were invited to pay a visit to the Longhua District Center for Disease Control and Prevention (CDC). Well-trained fieldworkers briefed prospective participants about the study and confirmed their eligibility to participate in the study. Guarantees were made on anonymity and their right to quit at any time, and that refusal would have no consequence. Written informed consent was obtained. Of the 2,700 workers in the selected workshops, 2023 completed a self-administered questionnaire which took about 30 min to complete. The response rate was 75%. Upon completion of the survey, a cash coupon of ¥20 (US2 0.60) was given to each participant for their time spent. Since we focused on migrant workers, local workers and those who did not report their homeland were excluded; 1,805 migrant workers were included for data analyses in this study.

### Measurements

Job stress was measured by the 13-item Job Stress Questionnaire (Caplan et al., 1975). The scale assesses four dimensions of job stress, including workload, role conflict, role and dignity, and utilization of skills. Items are rated on a 7-point Likert scale (1 = never to 7 = always). Higher scores mean stronger perceived job stress. The scale has been used in Chinese populations (Yu et al., 2009; Liu et al., 2015). The scale reliability was excellent in the current study (Cronbach’s alpha = 0.90).

Psychological adaptation was measured by the brief psychological adaptation scale (Demes and Geeraert, 2014). The items were adapted into the context of Shenzhen. Sample items included “Excited about being in Shenzhen” and “Out of place, like you don’t fit into Shenzhen culture” (reverse-scored item). Items are rated on a Likert scale (1 = never to 7 = always). Higher scores mean higher levels of psychological adaptation. The Cronbach’s alpha was 0.79.

Depressive symptoms were measured by the 10-item Center for Epidemiologic Studies Depression Scale (CESD-10) (Amtmann et al., 2014). It is a short version of the CESD-20 and has good reliability and validity (Amtmann et al., 2014). A cut-off point of ≥10 denotes probable depression; it was predictive of depression diagnosis (Arias et al., 2007; Björgvinsson et al., 2013). Items were rated on 4-point Likert scales, ranged from 0 (less than 1 day) to 3 (5–7 days). Higher scores indicate greater depressive symptoms. The Chinese version of the scale was validated in the Hong Kong population (Cheung and Bagley, 1998). The scale reliability was good in the current study (Cronbach’s alpha = 0.83).

Anxiety symptoms were measured by the 7-item Generalized anxiety disorder (GAD-7) scale (Spitzer et al., 2006). The GAD-7 was developed based on DSM-IV criteria and is used to measure the severity of generalized anxiety disorders in the past 2 weeks. It has also been shown to be a reliable screening tool for panic, social anxiety and post-traumatic stress disorder. Participants respond on a 4-point Likert type scale, from 0 (none) to 3 (almost every day). The cutoff point of probable anxiety is 5. The Chinese version has been validated in previous studies (Tong et al., 2016). The scale reliability was excellent in the current study (Cronbach’s alpha = 0.92).

Internet gaming disorder was measured by the 9-item DSM-5 IGD symptoms checklist (American Psychiatric Association [APA], 2013). It is a short, user-friendly, self-reported measure assessing IGD symptoms of preoccupation, tolerance, withdrawal, unsuccessful attempts to limit gaming, deception or lies about gaming, loss of interest in other activities, use despite knowledge of harm, use for escape or relief of negative mood, and harm based on DSM-5 criteria (American Psychiatric Association [APA], 2013). Response options include no (0) and yes (1). A score of 5 is taken as the cutoff point for defining IGD. The Chinese version was found to have good psychometric properties in Chinese populations (Ko et al., 2014; Yang et al., 2021). The scale reliability was excellent in the current study (Cronbach’s alpha = 0.90).

### Statistical Analysis

Descriptive statistics, including frequency, means and standard deviations (SD), were computed for participants’ background and psychological status. Simple and multiple linear/logistic regression analyses were conducted to test the associations between background/psychological variables and IGD. Structural equation modeling (SEM) was conducted to test the proposed mediation model. The indicators of job stress and psychological adaptation were created either based on scale dimensions or using random parceling approach. The mean scores of depression and anxiety were calculated and used as two indicators of negative affective states. Goodness of fit was tested by using the χ2 test, the Comparative Fit Index (CFI), the Non-normed Fit Index (NNFI), and the Root Means Square Error of Approximation (RMSEA) (Hu and Bentler, 1999). Standardized path coefficients (β) were reported. Bootstrapping analyses tested the mediation hypotheses. The 95% confidence intervals (CI) of the indirect effects would be obtained from 5,000 bootstrap samples. A statistically significant mediation effect would be observed when the CI did not include zero. The level of statistical significance was 0.05, and SPSS version 21.0 and Amos Version 26 were used for data analyses.

## Results

Table 1 presents participants’ background characteristics. Most of the participants (67.3%) were male, 35 years old or below (71.7%), came from villages (69.6%), had high school education or below (91%), and had monthly income of 4,999 RMB or below. About half of them (46.8%) had lived in Shenzhen for 2–10 years. Of the participants, 39.3, 28.7, and 7.5% were classified as having probable depression, probable anxiety, and IGD, respectively.

**TABLE 1:** Background characteristics of the participants (*N* = 1805).

Background Characteristics	*n*	%
**Sex**		
Male	1214	67.3
Female	591	32.7
**Age**		
<26	378	21.0
26–30	437	24.2
31–35	478	26.5
>35	489	27.1
Missing	13	0.8
**Hometown**		
Large city	36	2.1
Middle/small city	282	14.7
Village	1253	69.6
Missing	234	13.6
Education		
Middle school or below	1059	58.6
High school	585	32.4
College or above	113	6.3
Missing	48	2.7
**Monthly Income (RMB)**		
<3000	166	9.2
3000–4999	1099	60.9
>4999	484	26.8
Missing	56	3.1
**Years in Shenzhen**		
<2	495	27.4
2–5	527	29.2
6–10	318	17.6
>10	431	23.9
Missing	34	1.9

As Table 2 showed, sex, age, and years in Shenzhen significantly affected IGD scores. Males, younger people, and those who had lived in Shenzhen for fewer years showed greater IGD scores. All the psychological variables were significantly correlated with each other (Table 3).

**TABLE 2:** Internet gaming disorder (IGD) scores by background status.

Background variables	IGD scores		
	Mean ± SD	F(df)/t(df)	p
Sex		10.15	**<0.001**
Male	1.06 ± 2.08	(1919.5)	
Female	0.29 ± 1.28		
Age		12.71	**<0.001**
<26	1.18 ± 2.15	(3,2003)	
26–30	0.81 ± 1.84		
31–35	0.85 ± 1.97		
>35	0.44 ± 1.54		
Hometown		0.43	0.65
Large city	0.93 ± 1.92	(2,1744)	
Middle/small city	0.71 ± 1.78		
Village	0.80 ± 1.90		
Education		0.47	0.63
Middle school or below	0.77 ± 1.86	(2,1962)	
High school	0.86 ± 1.93		
College or above	0.82 ± 1.92		
Monthly income		0.53	0.59
<3000	0.94 ± 2.05	(2,1951)	
3000–4999	0.79 ± 1.89		
>4999	0.78 ± 1.84		
Years in Shenzhen		3.7	**0.01**
<2	0.81 ± 1.83	(3,1980)	
2–5	0.98 ± 2.05		
6–10	0.79 ± 1.92		
>10	0.60 ± 1.74		

*SD = standard deviation.*

*Bold value means a p-value less than 0.05.*

**TABLE 3:** Bi-variate correlations between psychological variables and IGD.

	1	2	3	4
1 Job stress	1			
2 Psychological adaptation	−0.35**	1		
3 Depressive symptoms	0.15**	−0.32**	1	
4 Anxiety symptoms	0.21**	−0.28**	0.65**	1
5 IGD	0.11**	−0.09**	0.21**	0.21**

**Correlation is significant at the 0.01 level (2-tailed).

*SD = standard deviation*

**FIGURE 1:**
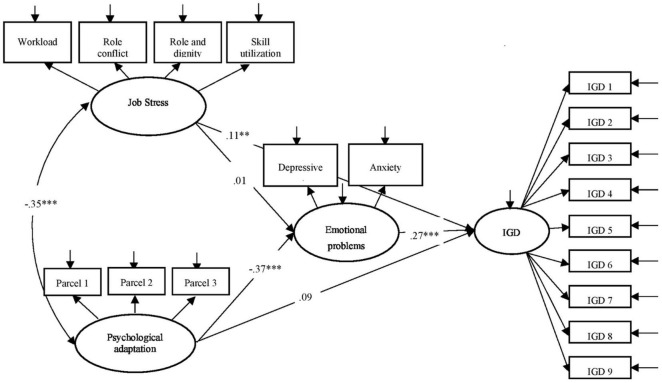
The proposed meditation model with standardized path coefficients.

The measurement model showed good model fit, χ2(df) = 1075.54(129), *p* < 0.05, CFI = 0.93, NNFI = 0.92, and RMSEA = 0.06. The structural model (Figure 1) fitted the data well, χ2(df) = 1075.53(129), *p* < 0.05, CFI = 0.93, NNFI = 0.93, and RMSEA = 0.06. Job stress was significantly associated with IGD (*B* = 0.03, β = 0.11, *p* = 0.01) but not with negative affective states (*B* = 0.03, β = 0.01, *p* = 0.77). Psychological adaptation was significantly associated with negative affective states (*B* = −2.93, β = −0.37, *p* < 0.001) but not with IGD (*B* = 0.04, β = 0.09, p > 0.05). Negative affective states were positively associated with IGD (*B* = 0.02, β = 0.27, *p* < 0.001). The indirect effect of psychological adaptation (*B* = −0.05, β = −0.10, 95%CI = −0.14 to −0.07, *p* = 0.004) but not job stress (*B* = 0.001, β = 0.003, 95%CI = −0.02 to.03, *p* = 0.77) on IGD through negative affective states was statistically significant. The significant indirect effect and insignificant direct effect of psychological adaptation on IGD suggested a full mediation effect of negative affective states.

## Discussion

The current study represents the first attempt to investigate the prevalence of IGD and related factors in migrant factory workers in China. The prevalence of 7.5% is higher than that in blue collar (6.1%) and white collar (5.5%) reported in a previous Chinese study (Chen et al., 2012) and that in Chinese community adults (2.0–2.6%) (Wu et al., 2018; Wang and Cheng, 2021). It may suggest that the study population is a high-risk group of IGD and interventions for IGD reduction for this population are warranted. Particular attention should be paid to and intervention effort should be made for males, younger people, and new migrants as they showed higher IGD scores than their counterparts. It may be because younger people and new migrants may have low social capital and social support which may increase their risk of maladaptive coping and behavioral problems (Worby and Organista, 2007; Wong and Leung, 2008; Qiu et al., 2011).

Furthermore, we identified significant psychological correlates of IGD among migrant factory workers. This is the first study that investigated the relationships between psychological adaptation/job stress and IGD. First, psychological adaptation was negatively associated with IGD. Indeed, psychological adaptation is related to a state of happiness with respect to being in the new culture which can protect migrant factory workers from emotional problems and maladaptive behaviors, while maladaptation may be a key source of stress for this population (Wong and Chang, 2010; Qiu et al., 2011). Besides psychological adaptation, other types of adaptation, such as sociocultural adaptation (i.e., the practical and behavioral aspects of adapting to a new culture), may also affect how a person navigate the culture effectively on a day-to-day basis and stress coping responses. Future studies may explore the roles of different types of adaptation in affecting IGD to better understand protective factors of IGD and other maladaptive behaviors among migrant factory workers.

Second, we found a positive correlation between job stress and IGD in migrant factory workers. Previous studies on stress and IGD were often conducted among student populations and found that general stress and specific stress (e.g., academic stress) were significant risk factors of IGD (Yu et al., 2018; Canale et al., 2019; Jeong et al., 2019). The present study is the first study that was conducted in a working population and tested job stress as a potential factor of IGD. Job stress can be a chronic condition and may be particularly salient for migrant factory workers as they are often lack of coping resource. Other types of stress, such as solitude (Yang et al., 2009) and disposable income (Chen et al., 2004), have also been implicated as risk factors of behavioral problems (e.g., smoking, drug use) in migrant populations. These potential factors should be investigated in future work on IGD to better understand its factors among migrant factory workers.

Importantly, potential mechanisms between job stress/psychological adaptation and IGD were explored based on the framework of general strain theory (Agnew, 1992). We found a significant full mediation effect of negative affective states on the association between psychological adaptation and IGD, which highlights the importance of affective status in this association. In other words, a lack of psychological adaptation can cause negative affective states and IGD may be a consequence of excessively adopting Internet gaming to regulate or escape from such states (Blasi et al., 2019). Similarly, one previous study based on the same theory demonstrated that depression significantly mediated the relationship between perceived academic stress and Internet addiction among adolescents (Jun and Choi, 2015). This is the first attempt to extend the general strain theory (Agnew, 1992) to explain how and why migrant factory workers may develop IGD. As such, one plausible extension of the theory could include an expansion of the criterion space to incorporate other social groups, types of strain and behavioral problems. It highlights the importance of understanding the complex interplay between strain related to cultural/environmental change and how a person processes and regulates such strain which could result in differential behavioral outcomes.

However, job stress was not significantly associated with negative affective states in the mediation model, resulting in an insignificant mediation effect of negative affective states between job stress and IGD. Considering the significant correlation between job stress and negative affective states in bi-variate correlation analyses but not in SEM and the strong covariance between job stress and psychological adaptation, we speculate that job stress and psychological adaptation may share some common variances in explaining the development of negative affective states. Thus, job stress became insignificant when psychological adaptation was added in the model. Indeed, pressures related to work and adaptation in a new culture may reciprocally affect each other and fuel a cycle of distress for migrant factory workers. A study has reported a positive correlation between adaptation and job related wellbeing among immigrants (Neto et al., 2019). Longitudinal studies are warranted to examine how they dynamically affect one another and conjunctively influence emotional health among migrant factory workers. Some potentially important mediators between job stress and IGD need to be further explored in future work. For example, great workload may lead to work-life imbalance and unhealthy lifestyle (Cheung and Yip, 2016; Zhang, 2020), including excessive Internet gaming. Other dimensions of job stress, such as role and dignity as well as utilization of skills, may be associated with one’s self-esteem and self-efficacy which may further influence IGD.

The findings have important practical implications. Migrant factory workers may be a high-risk group of IGD because they suffer from the dual sources of pressures, including those from job and adaptation in a new culture and Internet gaming is relatively inexpensive and handy for this population to escape from stressful reality and emotional problems such as depression and anxiety. Health promotion for migrant factory workers is greatly warranted but has been overlooked by mental health professionals and policymakers in China. Our preliminary findings suggest that early prevention of negative affective states and stress may be helpful in halting the development of IGD in this population. Stress reduction interventions and cognitive-behavioral therapies that teach individuals to manage stress and to learn emotion regulation skills may benefit these workers to prevent IGD. For example, an intervention study, based on the general strain theory, provided stress management training as one of its components and reported significant reduction in students’ academic stress and Internet addiction (Busari, 2016). It is also important to make recommendations about the adaptive stress coping and emotion regulation strategies for migrant factory workers to prevent and reduce their negative affective states and perceived stress. Physical activity may be an alternative and feasible option for these workers to regulate their emotions and coping with their stress (Liu et al., 2015). In addition, factories in Shenzhen are mostly built in isolated and remote areas to save cost which does not a friendly environment for migrants’ successful adaptation. Thus, creation of supportive environment and social networks for promoting social support and cultivating healthy lifestyle may facilitate adaptation in a new culture/environment and reduce the dependence of Internet gaming in this population. Further needs assessments and pilot interventions are in great need to design such intervention programs. Last but not least, factory managers should recognize the high prevalence of negative affective states and the risk of IGD among their employees and potential negative implications of such problems onto their health and subsequent productivity. Policies and regulations should be made to enhance factory managers’ awareness of the importance of factory workers’ health and health risks and to highlight their responsibility in promoting factory workers’ wellness.

The major limitation of this study is that the cross-sectional design could not address the causal effects of job stress/psychological adaptation on IGD and the mediation effect of negative affective states. It is plausible that IGD may increase emotional and interpersonal problems, cognitive impairment, and other behavioral problems, which can negatively affect job performance and increase difficulties in adaptation in a new culture. Thus, longitudinal studies to understand their dynamic relationships are warranted. Second, IGD was measured by self-reported without clinical diagnosis. Common method biases might have an influence on the prevalence of IGD. It is also possible that participants might respond in socially desirable ways that can interfere with the accurate interpretation of the data. Peer assessments may offer important and different perceptions to enhance our understanding of Internet gaming behavior in this population.

To conclude, sex, age, and years in Shenzhen, job stress and psychological adaptation were significantly associated with IGD scores. Furthermore, psychological adaptation was indirectly associated with IGD through negative affective states, while job stress was positively and directly associated with IGD. This study provides preliminary evidence for applying the general strain model to understand IGD among migrant factory workers. Effort to prevent negative affective states and IGD as well as other risk behaviors in this special and huge population in China is warranted. The identified background and psychological correlates may be helpful to design tailored interventions for this population.

## Data Availability Statement

The raw data supporting the conclusions of this article will be made available by the authors, without undue reservation.

## Ethics Statement

The studies involving human participants were reviewed and approved by the Ethics Committee of School of Public Health, Sun Yat-sen University (2019/3) and conducted in accordance with the Declaration of Helsinki. The patients/participants provided their written informed consent to participate in this study.

## Author Contributions

HC, KZ, YoC, BC, HZ, ZW, and XY: conceptualization. HC, KZ, ZW, and XY: methodology and formal analysis. DY, KZ, YaC, TH, DC, LL, and SW: investigation and data curation. HC, KZ, HZ, ZW, and XY: writing – original draft preparation. DY, YoC, BC, HZ, ZW, and XY: writing—review and editing. HC, KZ, and ZW: project administration and supervision. All authors have read and agreed to the published version of the manuscript.

## Conflict of Interest

The authors declare that the research was conducted in the absence of any commercial or financial relationships that could be construed as a potential conflict of interest.

## Publisher’s Note

All claims expressed in this article are solely those of the authors and do not necessarily represent those of their affiliated organizations, or those of the publisher, the editors and the reviewers. Any product that may be evaluated in this article, or claim that may be made by its manufacturer, is not guaranteed or endorsed by the publisher.

## Abbreviations

IGD: Internet gaming disorder
DSM-5: the fifth edition of the diagnostic and statistical manual of mental disorders
ICD-11: the 11th revision of the international classification of diseases
CDC: center for disease control and prevention
CESD-10: the 10-item center for epidemiologic studies depression scale
SEM: structural equation modeling
CFI: comparative fit index
NNFI: non-normed fit Index
RMSEA: root means square error of approximation.
